# Ovariotomy on the Bitch1Read before the Students’ Veterinary Medical Society.

**Published:** 1899-02

**Authors:** John Corrigan

**Affiliations:** Student, Ontario Veterinary College


### OVARIOTOMY ON THE BITCH.[1]

By John Corrigan,
STUDENT, ONTARIO VETERINARY COLLEGE.

This subject is of much importance in some localities on account of bitches being taxed five dollars and dogs but one dollar. Spayed bitches are taxed the same as dogs, consequently there is much spaying to be done in the spring or about taxing time.

The following is the mode of operation as performed by Dr. E. F. Vorhis, of Owego, N. Y., with whom I practised last summer:

[1] Read before the Students' Veterinary Medical Society.

Prepare the animal by fasting over night, and when ready to operate give a hypodermic injection of morphine, one-quarter to one grain, according to the size and age of the animal. It takes about twenty minutes for the drug to take effect, and during that time I get the instruments aseptic and everything to operate. Wash the abdomen with warm water and castile soap, after which shave the part where incision is to be made; usually half way between the umbilical opening and the pubis in the median line. Suspend the animal by the hind legs, as this causes the intestines to settle down below the seat of operation, and wash thoroughly with a solution of creolin and water. Make an incision about an inch long through the skin and muscles only, in the median line, just in front of the symphysis pubis. Then make a careful incision through the peritoneum, and usually the cornua of the uterus are in plain view; but, if not, they can be brought into sight with a curved probe, inserted into the incision. Make sure that this is not a portion of the intestine. To be sure of this follow the cornu up to the body of the uterus, where, at its junction, the other cornu will be found. Ligate both cornua, about one-half inch from the body of the uterus, with catgut. Follow each cornu to its respective ovary and remove it. Cut off the cornua near where they are ligatured. Sew up the peritoneum and muscles with catgut, and the skin with silk, and the operation is complete.

Wash the parts again with the creolin solution, and lay the animal in some quiet place to recover from the effects of the morphine. No after-treatment is needed. Caution the owner of the animal to be careful about feeding heavily for a few days, and to take out the external stitches.

We operated on at least fifty cases last summer, and all terminated most favorably. From the results of my experience in ovariotomy in the bitch, I feel safe in recommending this mode of operation, and I can further say that I strongly approve of the use of morphine hypodermatically as an anæsthetic, it having none of the bad results that occasionally ensue from the use of ether or chloroform on the dog.

---

A Rubefacient Soap.—℞.—Common white soap in shavings, 1 pound; dissolve in a quart of hot waetr, then add oil of turpentine, 1 pint, in which has been dissolved 2 to 4 ounces of gum camphor. An excellent embrocation in inflammatory affections around the joints of cattle.

